# Imaging of Chlorophyll *a* Fluorescence in Natural Compound-Induced Stress Detection

**DOI:** 10.3389/fpls.2020.583590

**Published:** 2020-12-21

**Authors:** Adela M. Sánchez-Moreiras, Elisa Graña, Manuel J. Reigosa, Fabrizio Araniti

**Affiliations:** ^1^Department of Plant Biology and Soil Science, Faculty of Biology, University of Vigo, Vigo, Spain; ^2^CITACA, Agri-Food Research and Transfer Cluster, University of Vigo, Ourense, Spain; ^3^Department AGRARIA, University “Mediterranea” of Reggio Calabria, Reggio Calabria, Italy

**Keywords:** plant natural compounds, phytotoxicity, imaging fluorescence, photosynthesis, plant stress

## Abstract

Imaging of chlorophyll *a* fluorescence (CFI) represents an easy, precise, fast and non-invasive technique that can be successfully used for discriminating plant response to phytotoxic stress with reproducible results and without damaging the plants. The spatio-temporal analyses of the fluorescence images can give information about damage evolution, secondary effects and plant defense response. In the last years, some studies about plant natural compounds-induced phytotoxicity have introduced imaging techniques to measure fluorescence, although the analysis of the image as a whole is often missed. In this paper we, therefore, evaluated the advantages of monitoring fluorescence images, presenting the physiological interpretation of different possible combinations of the most relevant parameters linked to fluorescence emission and the images obtained.

## Introduction

Considered as a non-invasive, fast and cheap technique that allows to rapidly detect and localize stressors effects on plants, chlorophyll *a* fluorescence (CF), has been largely used in plant science in the last decades, both clamping- and image-based Chlorophyll *a* fluorescence analysis (CFA) (Dayan and Zaccaro, 2012; de Carvalho et al., 2016). CFA gives the possibility to estimate, almost instantly, the photosynthetic efficiency under stress conditions and is especially useful in the detection of early stress responses (Chaerle and van der Straeten, 2000; Chaerle and Van Der Straeten, 2001; Chaerle et al., 2009). That is the reason why fluorescence measurement has been increasingly used to measure phytotoxicity in the last years.

Considering that herbicides will continue to be the worldwide method of weed control, easily and reproducibly exploring the wide range of molecules that nature offers seems the most appropriate way to search for new eco-herbicides. Secondary plant metabolites with relevant herbicide potential are essential in this context (Duke et al., 2000b), as the use of natural products allows protecting *a priori* the quality of the environment and, at the same time, the health of mammals during the control of weeds (Liebman, 2001). Therefore, in recent years, the study of the mode of action of natural compounds with herbicide potential is occupying a preferential place in integrated agriculture, thanks to the new physiological and molecular techniques that can integrate its multidisciplinary study (Sánchez-Moreiras et al., 2018). Among the several reasons for a long period without new herbicide target sites is also the difficulty to find new effective modes of actions for killing plants (Duke, 2011).

Looking at natural compounds, which have been undergoing strong evolutionary pressure for hundreds of years, would help to give light on this aspect. In fact, precisely the mode of action of these molecules is one of the potential advantages of secondary metabolites over synthetic herbicides. Natural compounds frequently present a strong structural diversity, which could result in novel molecular sites of action different from those already known for commercial herbicides. This would allow facing the weed resistance problem with a broader arsenal of modes of action (Duke et al., 2000a). The use of natural products as agrochemical compounds must necessarily go through the characterization of their mechanisms of action to know where, when and how they act in plant metabolism. That is the ultimate objective. However, a truly low number of modes of action is known at the current stage of research in plant natural compounds, which complicates their study and classification. Therefore, most studies on phytotoxicity induced by plant secondary metabolites focus on monitoring of their effects from a physiological perspective to detect the early effects of the compounds on roots and shoots. In this sense, CFA analysis is one of the techniques that could easily show the effects of bioherbicides on leaves. As stated by Dayan and Zaccaro (2012), the crucial step in the discovery of natural products that could be acting as photosystem II (PSII) inhibitors is simply evaluating the photosynthetic electron transport activity in leaves. Precise and reproducible CFA provides the demanded detailed analysis of the photosynthesis inhibition.

During the last 20 years, some excellent papers reviewed the use of CFA to measure stress response in plants (Maxwell and Johnson, 2000; Nedbal and Whitmarsh, 2004; Papageorgiou and Govindjee, 2004; Roháček et al., 2008; Kalaji et al., 2014, 2017; Guidi et al., 2019), while others focused on the use of imaging techniques to establish stress-related changes in chlorophyll fluorescence parameters (Chaerle et al., 2009; Martínez-Peñalver et al., 2011; Gorbe and Calatayud, 2012; Guidi et al., 2016). In this paper, we will provide a review of studies that applied chlorophyll fluorescence imaging (CFI) to monitoring the phytotoxic effects induced by plant natural compounds. Furthermore, we will present the physiological interpretation of possible results obtained by CF recording and will highlight the importance of considering both the PSII efficiency and its distribution over the plant.

## Chlorophyll Fluorescence Analysis: A Simple Method for a Complex Picture of PSII

The principles of chlorophyll fluorescence analysis are relatively simple. Basically, absorption of a photon promotes an electron of a chlorophyll *a* molecule to an excited state while a fluorescence photon is immediately emitted again during the return of the molecule to the ground state.

The first observation of fluorescence induced by solar radiation was recorded almost two centuries ago by David Brewster, which observed that illuminating a laurel leaves alcoholic extract with a sunbeam elicited a brilliant red light (Brewster, 1834). Moreover, he also observed that passing through the extract, the emission changed its color varying from red to orange and then to yellow, suggesting for the first time that this transition was probably due to chlorophyll re-absorption (Govindjee, 1995). The term “chlorophyll fluorescence” was coined by Stokes (1852) to describe this emission. Successively, Müller (1874) suggested a link between fluorescence emission and photosynthetic assimilation, which was successively confirmed by Kautsky and Hirsch (1931). These authors described, for the first time, the kinetics of chlorophyll *a* fluorescence emission of leaves previously adapted to dark, and then suddenly irradiated with light, correlating the signature of the initial fluorescence peak and its prompt decay to a lower steady-state to the time course of CO_2_ assimilation (for details see Govindjee, 2004).

The covariation between photosynthesis and chlorophyll fluorescence was successively described by McAlister and Myers (1940). They reported and described two different processes: (i) one characterized by an inverse relation between CF intensity and the rate of CO_2_ uptake, and the other (ii) considering a direct relationship between these two parameters.

A detailed description of this dual response was successively described by Duysens and Sweers (1963), which used a modulated excitation light for the first time and described, also for the first time, that the fluorescence yield was actively regulated by a process known today as “non-photochemical quenching” (Weis and Berry, 1987; Krause and Weis, 1991). This information was successively used to prove a quantitative relationship between the electron transport rate and the fluorescence yield (Weis and Berry, 1987; Genty et al., 1989).

After that, it became clear that CF is one of the mechanisms for energy dissipation, together with photochemistry and non-photochemical quenching (Schreiber, 1983; Krause and Weis, 1984; Demmig-Adams et al., 1990; Havaux et al., 1991; Björkman and Demmig-Adams, 1995). Therefore, being able to relate CF with changes in the photosynthetic apparatus it allows the quantification of the extent to which CF decreases by photochemistry (namely photochemical quenching) or non-radiative decay (non-photochemical quenching).

From the 1990s on, CFA has been widely used with the attempt to detect modifications occurring in the photosynthetic process, especially in plants under stress conditions (Schreiber and Neubauer, 1987; Endo et al., 1995; Maxwell and Johnson, 2000; Papageorgiou and Govindjee, 2004). CF parameters are very sensitive and detect the emergence of stress, even before visible symptoms appear over the leaf lamina, or a decline of photosynthesis can be determined by gas exchange measurements (Gorbe and Calatayud, 2012). However, in some cases, i.e., in plants subjected to abiotic stressors leading to rapid stomata closure, CF parameters might have a delayed response when compared to gas exchange analyses (Schreiber et al., 1995).

CF can be excited by illuminating green plant tissues with pulsed (Schreiber et al., 1986; Schreiber, 2004) or continuous (Strasser and Govindjee., 1991) visible or ultra violet (UV) light by either single or grouped LEDs (light emitting diode) or halogen lamps. In addition, deuterium lamps were also used in old systems (Schreiber et al., 1993). Using visible light, light-induction kinetics, as well as the measurement in the true steady state, is one of the most widely used approach in CF measurement (Lazár, 2003; Gorbe and Calatayud, 2012). This technique, which uses actinic light illumination, allows monitoring and measuring both the photochemical and non-photochemical processes involved in fluorescence quenching. The pulse amplitude modulated (PAM) fluorometer is the most common and largely used fluorometer in CF monitoring (Bolhar-Nordenkampf et al., 1989; Häder et al., 1998). In addition, also OIJP test is a very informative and routinely used method to evaluate fast kinetics of CF (Hermans et al., 2003; Digrado et al., 2017).

Depending on the state of electron transport chain components and dark/light adaptation of the photosynthetic tissues, five different CF emission signals can be detected: *F*_0_, *F*′_0_, *F*_*m*_, *F*′_*m*_, and *F*_*s*_ (Maxwell and Johnson, 2000) (Table 1). *F*_0_ and *F*_*m*_, determined on dark-adapted samples, correspond to both minimum and maximum fluorescence yield before and after a saturating light pulse takes place, respectively. Briefly, a period of dark adaptation (20–30 min) allows all reaction centers to be open and the exposure to modulated weak light (<1.0 μmol photons m^–2^ s^–1^ which is not sufficient to induce an electron transport chain) induces an increase in the CF yield, namely *F*_0_ (Table 1). Then, a strong saturating light pulse (from 8,000–15,000 μmol photons m^–2^ s^–1^ which induces the closure of all reaction centers) is applied and induces a rapid increase in the CF yield, namely *F*_*m*_ (Table 1). After that, CF yield decreases and, in the presence of actinic light, it reaches steady-state values (*F*_*s*_) that correspond to a balance between the reduced and oxidized state of primary electron acceptor *Q*_*A*_. Conversely, *F*′_0_ and F′_*m*_ are determined after sample receives actinic illumination, and represent the minimal and maximal fluorescence yield in light conditions, respectively (Table 1). The determination of F_0_′ under light conditions requires the use of far-red light to transiently and selectively excite PSI, and thus to enhance the oxidation of the electron transport chain (Bilger and Schreiber, 1987).

**TABLE 1:** List and definition of the most common chlorophyll fluorescence parameters.

**Fluorescence parameters**	**Definition**
*F*_0_	Known as dark fluorescence yield it is measured on dark-adapted plants (all PSII reaction centers are open) and represents the minimal chlorophyll fluorescence intensity.
*F*_*m*_	Also measured on dark-adapted plants during the application of a saturating pulse of light and represent the maximal chlorophyll fluorescence intensity.
*F*′*_0_*	Parameter measured during the light-adapted state and represents the minimal chlorophyll fluorescence intensity.
*F*′*_*m*_*	Parameter measured on light-adapted plants during the saturation pulse and representing the maximal chlorophyll fluorescence intensity.
*F*_*s*_	Parameter measured during the steady-state, non-saturating actinic illumination, representing the chlorophyll fluorescence intensity
*F*_*v*_ = (*F*_*m*_-*F*_0_)	Parameter measured during the dark-adapted state (non-photochemical processes are at the lowest level) representing the variable chlorophyll fluorescence
*F*′*_*v*_*	Parameter *F*_*v*_ measured during the light-adapted state
*F*_*v*_/*F*_*m*_	Maximum quantum yield of dark adapted PSII
*F*′*_*v*_*/*F*′*_*m*_*	Parameter measured during the light-adapted state representing the exciton transfer efficiency from antenna pigments to PSII reaction centers
*F*_0_/*F*_*v*_	Parameter measured during the dark-adapted state representing the Ratio of minimal chlorophyll fluorescence intensity/variable chlorophyll fluorescence
*F*_*m*_/*F*_*s*_	Parameter that indicates the photosynthetic quantum conversion obtained from ratio between the maximal chlorophyll fluorescence intensity (measured in the dark-adapted state) and chlorophyll fluorescence at steady-state.
*RF*_*d*_ = (*F*_*m*_–*F*_*s*_)/*F*_*s*_	This parameter (*R*_*Fd*_ – chlorophyll fluorescence decreases ratio), correlated with CO_2_ fixation rates, is an indicator of the photosynthetic quantum conversion.
NPQ = (*F*_*m*_–*F*′*_*m*_*)/F′*_*m*_*	Stern-Volmer’s non-photochemical quenching coefficient
Φ*_*II*_* = (*F*′*_*m*_*–*F*_*s*_)/*F*′*_*m*_*	Effective PSII quantum yield
Φ*_*NO*_* = 1/[NPQ + 1 + qL (*F*_*m*_/*F*_0_-1)]	Quantum yield of non-regulated energy dissipation in PSII. High Φ*_*NO*_* values indicate that both protective regulatory mechanisms and photochemical energy conversion are inefficient, suggesting that plant is unable to cope with the incident radiation.
Φ*_*NPQ*_* = 1-Φ*_*II*_*-Φ*_*NO*_*	Quantum yield of regulated energy dissipation in PSII representing the ability of PSII to dissipate the energy in excess in the form of heat
Φ*_*NPQ*_*/Φ*_*NO*_*	Parameter representing the ratio of quantum yield of regulated to non-regulated energy dissipation in PSII. It is connected to the ability of photosynthetic apparatus to activate photoprotection mechanisms
q*N* = (*F*_*m*_–*F*′*_*m*_*)/(*F*_*m*_–*F*′*_0_*)	Parameter representing the coefficient of non-photochemical quenching of variable fluorescence
q*P* = (*F*′*_*m*_*–*F*_*s*_)/(*F*′*_*m*_*–*F*′*_0_*)	Parameter based on the puddle model of PSII representing the coefficient of photochemical quenching of variable fluorescence
q*L* = qP × *F*_0_/*F*_*s*_	Parameter based on the lake model of PSII representing the coefficient of photochemical quenching of variable fluorescence

*The table includes only the most common parameters, for comprehensive reviews on this topic refer to the review of Baker (2008) and Maxwell and Johnson (2000).*

Using these five fluorescence emission signals is possible to calculate other CF parameters. Among them, it is worth to mention the maximal PSII quantum yield (*F_*v*_/F_*m*_*), the effective PSII quantum yield (*Φ*_*II*_), both photochemical (q_*P*_) and non-photochemical quenching (NPQ), as well as the quantum yield of regulated (*Φ*_*NPQ*_) and non-regulated energy dissipation (*Φ*_*NO*_), which compete with *Φ*_*II*_ for energy partitioning (i.e., Φ_*II*_ + Φ_*NO*_ + Φ_*NPQ*_ = 1; Kramer et al., 2004) (Table 1). Of note, the q_*NP*_ coefficient consists of three different components: energy-dependent quenching related to the build-up of the trans-thylakoidal pH-gradient, *q*_*E*_; quenching due to the state II-I transition of the phosphorylated light harvesting complex of PSII, *q*_*T*_; and photo-inhibitory quenching, *q*_*I*_ (Horton and Hague, 1988).

Nowadays, other new parameters have been introduced with the attempt to make CF a very detailed diagnostic tool. In some cases some stress-related parameters, allow to anticipate the occurrence of visible symptoms over the leaf lamina. As an example qPd (photochemical quenching measuring in the dark) enables the detection of earliest signs of photoinhibition (when qPd value is 1 corresponds to 100% of open RCIIs) (Ruban and Murchie, 2012). In addition, the use of imaging-based instruments provides information on the distribution of CF over a selected area of the sample (Gorbe and Calatayud, 2012).

However, in view of the high number of excellent reviews on CF, both applicative and theoretical, the in-depth description of CF parameters is out of the scope of the present review (for example, for detailed reviews on CF parameters refer to Schreiber and Bilger, 1993; Schreiber et al., 1998; Maxwell and Johnson, 2000; Baker and Rosenqvist, 2004; Gorbe and Calatayud, 2012; Kalaji et al., 2014, 2017; Guidi et al., 2016).

## Tools for Imaging Chlorophyll Fluorescence

The invention of video imaging systems was an important breakthrough in the field of chlorophyll fluorescence analysis (Omasa et al., 1987; Fenton and Crofts, 1990). In fact, this technique not only allowed to spatially examine the heterogeneity within a sample, but it also made possible to evaluate those changes simultaneously on a wide number of samples, making the CFI more popular as diagnostic (Baker, 2008), screening (Barbagallo et al., 2003) and phenotyping tool (Rühle et al., 2018; Pérez-Bueno et al., 2019).

The use of chlorophyll photometer capable to image CFA is known from decades. At the beginning, the CFI instruments were able to image chlorophyll fluorescence at *F*_*m*_ and *F*_*m*_′ and at *F* or *F*_*s*_ by using a high incident irradiance, but those instruments were unable to image at *F*_0_, *F*_0_′, and *F*_*s*_ under moderate to high incident irradiance (Genty and Meyer, 1995; Siebke and Weis, 1995a,b; Scholes and Rolfe, 1996). Oxborough and Baker (1997) described the first instrument that was able to image those parameters. This instrument, mainly based on an epifluorescence microscopy connected to a CCD camera, was characterized by a large dynamic range, and by the capacity of generating images of CF using low incidence irradiance (0.1 μmol m^–2^ s^–1^). In addition, it gave the possibility to work on part of the leaf tissue as well as on single cells.

Lichtenthaler et al. (2005) developed a compact flash-lamp fluorescence imaging system, which was based on the Karlsruhe/Strasbourg laser-induced fluorescence imaging system (Lang et al., 1994; Lichtenthaler, 1996; Lichtenthaler and Miehé, 1997). The instrument was produced to replace, as excitation source, the expensive ND:YAG laser by a cheaper xenon flash lamp, which allows measurements with high pulse frequency and short gating times (Buschmann et al., 2000; Lichtenthaler and Babani, 2000; Lichtenthaler et al., 2005).

The technique, as well as the tools to monitor chlorophyll fluorescence emission, was further implemented and hyphenated with phenotyping tools for both diagnostic and breeding purposes.

CFI, associated to phenotyping tools, gives the possibility to early detect plants stress responses to both biotic and abiotic stressors, before the development of visible symptoms. At agricultural level, especially for sustainable/precision agriculture, the use of CFI as research tool could have an extensive field of application. CFI could facilitate the researchers in identifying and evaluating crop stress thresholds allowing, with the help of sensors (e.g., temperature, humidity and conductivity sensors among others), not only to make targeted and/or localized interventions but also to plan them only when necessary. This could permit to anticipate irreversible damages to the photosynthetic apparatus. Anyway, at the moment, CFI technique is too slow and expensive to be used as a commercial diagnostic tool in open field and/or greenhouses.

Depending on the crop and on the pedological and pedoclimatic conditions, it can be used as a research tool to extrapolate the stress-thresholds of a given species, which can be integrated in more complexes algorithms and used for crop management.

At both laboratory and field levels, Photon Systems Instruments (Brno, Czech Republic), Walz (Effeltrich, Germany), and Technologica Ltd., (Essex, United Kingdom) commercialized several instruments (for more details have a look at the website of the companies) that allow the application of this technology at macroscopic and microscopic levels, and on terrestrial and aquatic organisms (Levin et al., 2017; Ayalon et al., 2019).

Imaging fluorometers have been also integrated with a gas-exchange chamber, which allows to obtain information about CF, the imaging of its parameters, and also about the plant gaseous exchanges, giving wider information concerning the photosynthetic machinery status in response to stress (Daley et al., 1989; Rolfe and Scholes, 1995; Kurepin et al., 2018).

The combination of CFI with the technique of the infra-red gas exchange (IRGA) allows to directly correlating the efficiency of PSII with the CO_2_ assimilation rate. This could be achieved avoiding the photorespiration through the increase of CO_2_ concentration or reducing the concentration of O_2_. This technique has been used to visualize patterns of CO_2_ diffusion in leaves, enabling the determination of gas fluxes within leaves of different species (Morison et al., 2005, 2007; Lawson and Morison, 2006).

Also, the use of CFI tools in combination with other imaging techniques could give important information in plant phenotyping. For example, CFI coupled to hyperspectral imaging was used for early detecting fungal disease in wheat, whereas coupled to thermography it was used to provide information concerning the correlation between photosynthetic rate and stomatal behavior, as well as in imaging intrinsic water use efficiency (Chaerle et al., 2007; Lawson, 2009; Bauriegel et al., 2011).

Moreover, imaging fluorometers integrated with phenotyping platforms, which in the past were exclusively used for research purposes, are now commercially available (e.g., HyperAIxpert from Lemnatech; PlantScreen System from Photon Systems Instruments, etc.). These platforms, in addition to the phenotypic traits, allow imaging of chlorophyll fluorescence in order to get high-throughput analysis of plant phenotype (Awlia et al., 2016). To study plant-pathogen interactions as well as to investigate changes in plant physiology and biochemistry, in response to stress, imaging fluorometers have been integrated with a GFP filter for imaging (not simultaneously) green fluorescent proteins in transformed plants, and/or in plants inoculated with GFP-transformed strains of pathogens, which is extremely useful for molecular and cellular biology studies (Lee et al., 2019; Pérez-Bueno et al., 2019). Other imaging fluorometers have also been adapted to study the photosynthesis in plants growing under water, which allows to evaluate the response of plants to several stressors such as water pollution or CO_2_ variations (Papathanasiou et al., 2020).

Other tools, applicable to microscopy, have been also developed in order to allow the monitoring of CFI heterogeneities at the level of single cell (e.g., on algae or stomata guard-cells) and they could allow to taxonomically differentiate algae types, like diatoms, chlorophytes, and cyanobacteria (Oxborough and Baker, 1997; Levin et al., 2017).

For example, through the combination of a microscope CFI system equipped with an IRGA chamber, Lawson et al. (2002) were able to demonstrate that the major sink for guard cell photosynthetic electron transport was the Calvin cycle activity and that the photosynthetic efficiency of the guard cell is not linked to the opening and closing of the stomata.

The majority of CFI tools, to ensure a coverage of actinic illumination and fully saturating pulses on a huge portion of plant area, requires large panels on which light-emitting diode arrays are mounted making these tools extremely large in size and barely usable in open field. Nevertheless, some fluorescence monitoring tools, equipped with a cabinet (for dark-adaptation purposes) and mounted on wheels (to allow movement in the field collecting images over the crops), were built (Murchie and Lawson, 2013; Tan et al., 2018).

Finally, new generations of low-weight dfov (dual field of view) spectrometer systems have been developed to monitor and image solar-induced CF variations in both open fields and green houses. Those instruments can be managed remotely and located on both fixed and mobile workstations (i.e., drones) (Atherton et al., 2018).

## Imaging of Chlorophyll Fluorescence in the Study of Natural Compounds

The use of CF as a phenotyping and/or diagnostic tool was quickly growing in the last decade. Several reviews were written about it (Humplík et al., 2015; Pérez-Bueno et al., 2019) and new researchers are trying to extend its applications (Weber et al., 2017; McAusland et al., 2019).

Especially in herbicidal studies, CF has been extensively used as a marker for the study of herbicide mode of action or for the identification of weed resistance (Dayan and Zaccaro, 2012). However, although several manuscripts focused on both monitoring CF during several stress, including herbicides, and the pattern of fluorescence changes through imaging, relatively few information is available concerning the use of imaging signatures during the evaluation of the herbicidal potential of natural compounds and extracts.

Because of that, the best way to evaluate natural compounds target and mode of action should be to compare the fluorescence signature induced by natural compounds with that of the most known and used herbicides. Unluckily, the number of studies doing that is little, and the use of CFI tools in this field of the research is extremely low. Moreover, this technique is often used just to get PSII parameters without giving the right importance to the imaging output. As a consequence, the information concerning the pattern of action of natural phytotoxic compounds is fragmented and superficial.

One of the first studies, focusing on the application of CFI analysis on phytotoxic natural compounds, was reported by Beninger et al. (2004). In particular, they evaluated the phytotoxic potential of three phenolic compounds isolated from *Chrysanthemum morifolium* on the model species *Lemna gibba*. They observed that chlorogenic acid and the flavanone eriodyctiol strongly affected the photosynthetic activity with deep reductions in *F_*v*_/F_*m*_*. Both compounds acted as bleaching herbicides causing a loss of photosynthetic pigments and the inhibition of photosynthetic activity. However, interestingly, while overall *F_*v*_/F_*m*_* values were reduced just in the plants treated with 1000 ppm, the area of the plant experiencing photosynthesis was reduced also in the leaves treated with the lowest concentration (100 ppm), probably due to the loss of photosynthetic pigments.

Kriegs et al. (2010) demonstrated that the quantum yield efficiency (*F*_*v*_/*F*_*m*_) of Arabidopsis plants, fumigated with the monoterpene camphor, was directly affected by the treatment. In particular, they reported that during short-time fumigation the parameter *F*_*v*_/*F*_*m*_ was strongly lowered compared to control, but it promptly recovered at the end of the treatment, and the plant fitness was significantly strengthened. On the other hand, repeated exposure to the monoterpene led to irreversible damages and alteration in plant phenotype. Probably, the negative effects induced by long-term exposure could be connected to the high cuticular dewaxing ability of camphor as previously demonstrated by Schulz et al. (2007). However, although authors used the imaging apparatus Growscreen-Fluoro, an automated imaging pipeline designed to analyze the chlorophyll fluorescence of rosette plants, no images about the effects of this monoterpene on plants were shown in the manuscript. This is a pity since images could be extremely helpful in understanding the dynamic of the effects of the terpenoid and the overall plant responses to the treatment.

An example of how imaging techniques can broaden our knowledge on natural compound effects was published by Sánchez-Moreiras et al. (2011) with the allelochemical 2-3*H*-benzoxazolinone (BOA). Previous single-point measurements of photochemical and non-photochemical parameters in BOA-treated plants suggested that increase in fluorescence emission was due to BOA-induced oxidative stress (Sánchez-Moreiras and Reigosa, 2005). However, CFI together with imaging hydrogen peroxide and superoxide anion, revealed that the primary phytotoxic effect of BOA was the induction of premature senescence, and oxidative stress was just a secondary effect of this treatment (Sánchez-Moreiras et al., 2011).

The advantage of obtaining an image of CF and its distribution all over the plant in intact and alive individuals is one of the aspects that can most enrich the study of phytotoxic compounds, as it can gives information about the area of the leaf where the effect starts first. Studies carried out on the *trans*-cinnamic derivative *o*-coumaric acid, one of the major constituents of the invasive species *Eupatorium adenophorum*, demonstrated that this molecule significantly affected the photosynthetic machinery of the model species *Arabidopsis thaliana*, pointing out a differential effect on photosynthesis depending on the age of the leaves (Zheng et al., 2012). In particular, they observed that after 7 d of treatment, *F_*v*_/F_*m*_* dropped dramatically in older leaves, whereas younger central leaves were hardly affected, suggesting that *o*-coumaric acid could promote leaf senescence. In addition, Graña et al. (2013b) demonstrated that adult *Arabidopsis* plants sprayed with the monoterpenoid citral had a heterogeneous spatial distribution in fluorescence emission, mainly concentrated at the edges of the older leaves, whereas the youngest leaves were not affected. This could suggest the initiation of early senescence processes, where the photosynthetic apparatus is systematically dismantled (Wingler et al., 2004).

Several studies evaluating effects of natural products by CFI indicated that the majority of these products and/or their extracts generally reduced *F*_*v*_/*F*_*m*_. This may suggest a direct damage to PSII, whereas the yield of non-photochemical energy dissipation, *Φ*_*NO*_ and *Φ*_*NPQ*_, was extremely variable, probably depending on type and/or dose of the extract or compound assayed. In fact, natural compounds that are extremely phytotoxic at high doses can be stimulant at low doses, a phenomenon known as hormesis (Belz and Duke, 2017). For example, the aqueous extract of *Mentha X piperita* may be inhibitory or stimulatory on chlorophyll content and on PSII (analyzed by CFI) of sunflower leaves depending on the concentrations assayed (Skrzypek et al., 2015). The authors observed that low concentrations of peppermint extract significantly increased *F*_*v*_/*F*_*m*_ ratio and the *Φ*_*NPQ*_ on the entire leaf surface. In contrast, higher concentrations significantly reduced the regulated non-photochemical energy dissipation suggesting a negative effect on the photosynthetic machinery. Skrzypek et al. (2015) observed also that the highest changes in CF were specifically around the petioles and in the distal margins of leaf blade. Those results highlight the importance of imaging parameters, which can give a lot of quick and precise information of the exact stimulatory and inhibitory doses of a given compound, as well as the induced damage.

In adult chalcone-treated *Arabidopsis* plants, Díaz-Tielas et al. (2014) found an early decline in ETR concomitant with a significant increase in *Φ*_*NO*_ and a late reduction in maximum PSII efficiency (*F_*v*_/F_*m*_*). Supported by the imaging of damages localization, they suggested that the chalcone-induced reduction in photosynthetic rate could have resulted in an excessive demand on regulated antenna de-excitation processes inducing damages to the antenna complex and, consequently, *F_*v*_/F_*m*_* reduction. Based on these previous imaging studies, Díaz-Tielas et al. (2017) observed that chalcone treatment on young *Arabidopsis* seedlings induced progressive pigment degradation and bleaching. They demonstrated that this progressive de-greening, together with *F_*v*_/F_*m*_*, *Φ*_*II*_ reduction and *Φ*_*NO*_ increase, was directly linked to early plasma membrane depolarization and dramatic effects on chloroplasts structure and function, as part of chalcone mode of action.

Essential oils (EOs) are among the most studied and commercially used natural extracts with phytotoxic potential. Recent studies demonstrated that pure terpenoids, isolated from EOs, have a wide range of metabolic targets and modes of action (Graña et al., 2013a; Araniti et al., 2017a). It has been proven that several compounds can act synergistically changing completely the effects of these natural mixtures and/or pure molecules on the photosynthetic machinery. For example, Araniti et al. (2018a) demonstrated that EO isolated from *Origanum vulgare* inhibits glutamate and aspartate metabolism in *A. thaliana*. The inhibition of these metabolic pathways induced an accumulation of ammonia in leaf cells and, concomitantly, a cascade of reactions that limited the efficiency of PSII. Through the imaging of *F*_*v*_/*F*_*m*_ and *Φ*_*II*_ parameters authors were able to identify which areas of the leaves were mostly affected by EOs treatment. Moreover, they highlighted through the imaging output an overlap between reactive oxygen species (ROS) accumulation (monitored through *in situ* staining methods) and damages to PSII (reduction in *F*_*v*_\*F*_*m*_).

This was not the first work detecting overlap among PSII damage and ROS accumulation in response to terpenoids. Araniti et al. (2017b), studying plant-plant interaction, mimicking natural conditions, demonstrated that volatiles released by the widespread species *Dittrichia viscosa* were able to induce ROS accumulation in lettuce leaves causing *F*_*v*_/*F*_*m*_ inhibition. Through CFI, they also evidenced an overlap between the leaf area where ROS were accumulating and leaf area characterized by *F*_*v*_/*F*_*m*_ reduction. Those results suggested that probably the burning activity of the EOs could be mainly due to a side effect (ROS burst) instead of a direct contact of the chemical mixture with the leaves.

Recently, Synowiec et al. (2019) bioassayed the effects of caraway and peppermint EOs on *Zea mays* and its associated weeds. They demonstrated, through CFI, that the application of caraway EOs at a given concentration strongly affected the PSII of *Echinochloa crus-galli*. However, it was hardly effective on *Z*. mays, highlighting the species-specific effects of EOs, and suggesting that caraway EOs could be a good candidate for weed management in maize crop.

Graña et al. (2013b), bioassaying through sub-irrigation or spraying, the monoterpene citral on adult plants of *A. thaliana* observed that both treatments strongly affected either the photochemical or non-photochemical activity. Citral effects were earlier and more pronounced in sprayed plants than in watered. In fact, while sprayed leaves were severely damaged by the treatment, leaves of sub-irrigated plants were not apparently affected. ETR, *Φ*_*II*_, and *F*_*v*_/*F*_*m*_ values revealed that photosynthesis was reduced in citral-watered plants as a result of a general slowing down of the metabolism, while citral-sprayed plants were photoinhibited and presented physical damage due to the treatment. These results were also supported by the spatio-temporal CFI. The same experiments carried out by Araniti et al. (2017c), bioassaying the phytotoxicity of *trans*-caryophyllene on Arabidopsis adult plants, didn’t show any effects on plants sprayed, whereas PSII of plant sub-irrigated was extremely altered. They concluded that the observed PSII damage would be the consequence of plant water status alterations accompanied by ROS burst and oxidative stress. These results suggest that CFI outputs could be used in screening programs to highlight, depending on the molecule bioassayed, the best method of application of a chemical. In addition, concerning the last two chemicals (citral and *trans*-caryophyllene), the data further highlight the high potential of the imaging techniques as phenotyping tool, which can be helpful in giving hints concerning the target and the potential mode of action of natural phytotoxins.

CFI has been used also to evaluate the phytotoxic effects of natural compounds produced by fungi and bacteria. Guo et al. (2019) demonstrated that gliotoxin, a fungal secondary metabolite, affects both the electron transport through PSII at the acceptor side and the reduction rate of PSI end electron acceptors’ pool. Through CFI, authors identified the concentration inducing physical damage to PSII. In addition, Xiao et al. (2020) reported that alamethicin, an antimicrobial peptide isolated from fungus *Trichoderma viride*, acts on PSII with a mechanism similar to the commercial herbicide diuron (Laasch et al., 1983). In particular, this secondary metabolite would interrupt PSII electron transfer beyond the primary plastoquinone at the acceptor side, leading to PSII reaction centers inactivation. In addition, alamethicin destroys the PSII pigment architecture but does not affect the oxygen-evolving complex at the donor side. The damages in the leaves (necrotic areas on leaf surface) were visible only in plants treated with the highest concentrations of alamethicin, whereas the imaging output of the CFI system allowed to understand that even the lower concentrations altered the photosynthetic machinery (*F*_*v*_/*F*_*m*_ decrease) although damages were not evident, confirming again the power of this technique.

## Advantages and Limitations of CFI in Natural Products Stress Detection

Regarding the study of phytotoxicity induced by plant natural products, the majority of the works reported in literature, based on CF monitoring, were carried out with clamping tools that record single-point measurements on restricted leaf parts. The advantages of monitoring CF through imaging instead of single-point fluorescence have already been discussed (Oxborough, 2004; Lichtenthaler et al., 2005). Martínez-Peñalver et al. (2011) demonstrated that measurements obtained through single-point fluorescence do not always reflect the heterogeneity of the stress-related effects in plants. In their research, authors compared the temporal and spatial distribution of fluorescence emission in plants treated with allelochemicals, abiotic factors, and heavy metals. They observed that some stress factors, as the assayed heavy metals, induced a homogenous inhibition of the maximum PSII efficiency all over the whole plant, while in plants treated with allelochemicals the inhibition was mainly located on the margins of old leaves whereas younger leaves were unaffected. They suggested that these irregularities in CF distribution along plant make difficult any correlation with single-point measurements, typically done through clamping fluorometers.

Gao et al. (2018) bioassayed also the effects of usnic (UA), benzoic (BA), cinnamic (CA), and salicylic acid (SA) on the photosynthetic apparatus of the algae *Chlamydomonas reinhardtii*. They found that UA and SA have probably stronger photosynthetic inhibitory activity than CA and BA acids. The four phytotoxins showed multiple targets in chloroplasts. In particular, UA acid decreased photosynthesis inducing a reduction of PSII O_2_ evolution rate, interrupting PSII electron transport (strong ETR reductions), and inactivating the PSII reaction centers (strong *F_*v*_/F_*m*_* decrease). Based on the JIP-curve, UA-treated cells showed a fast increase of J-step, which is due to the accumulation of *Q*_*A*_^–^ when the electron flow beyond *Q*_*A*_ is blocked at the PSII acceptor side. The other site of action of UA would be the oxidizing site of PSII, as indicated by the low PSII O_2_ evolution rate. Moreover, UA reduced pigment content, damaging the conformation of antenna pigment assemblies, as suggested by the decreased values of ABS/CS (average antenna size per excited leaf cross-section) and TR_0_/CS (trapped energy flux per excited leaf cross-section), resulting in destroyed structure and function of PSII. Gao et al. (2018) suggested also that damages induced by SA on the photosynthetic machinery would be mainly attributed to inhibition of PSII electron transport beyond primary plastoquinone acceptor at the acceptor side, and the inactivation of the PSII reaction centers by decreasing Ψ_*EO*_ (the probability that an electron moves further than *Q*_*A*_^–^) and φ_*EO*_ (the quantum yield for electron transport). SA induced as well thylakoid membranes destabilization by changes at D1/D2 dimer and the polypeptides of the water-oxidizing complex. Finally, both CA acid and BA acid would act by reducing PSII electron transport efficiency at the acceptor side and the amount of active PSII reaction centers, but with some differences. In particular, BA disrupted the flow between antenna pigments and PSII reaction center, with decreases of *F*_*t*_, *F*_*M*_, *F_*v*_/F_*m*_*, and TR_0_/C, while CA did not affect these parameters, but decreased PSII O_2_ evolution rate.

Imaging techniques can be also decisive to study recovering processes. Plants treated with the allelochemical protocatechualdehyde (Martínez-Peñalver et al., 2012) highlighted tolerance to this molecule, as whole-plant images of *F_*v*_/F_*m*_* showed the greatest number of damage spots among 9 and 24 h after treatment, but images of 96, 144, and 192 h showed the recovering of PSII parameters within a week of treatment.

Recent studies carried on the phenolic acid *trans*-cinnamic acid, assayed at sub-lethal concentration, pointed out that, besides the reduction in growth, *trans*-cinnamic significantly stimulated the photosynthetic machinery of *Zea mays* seedlings (Araniti et al., 2018b). In fact, in plants treated with this known phytotoxic molecule, the dark adapted PSII parameter (*F_*v*_/F_*m*_*) was not affected, whereas a significant increment in pigment content, and the stimulation of the light adapted PSII efficiency (*Φ*_*II*_), the regulated dissipation of the energy in form of heat (Φ*_*NPQ*_*) and the apparent electron transport rate was observed. At the same time the CFA (Φ*_*NO*_*) was significantly stimulated by the treatment. Those data are supportive for the capability of the plant to cope with *trans*-cinnamic acid effects in different ways. From one hand, *Z. mays* plants increased the level of photochemistry and were able to increase the level of energy channeled toward the electron transport chain (see ETR and Φ*_*II*_* increments). On the other hand, treated plants showed also a higher capacity to dissipate the energy, which exceeds photochemistry through both regulated (see Φ*_*NPQ*_*) as well as unregulated mechanisms (see Φ*_*NO*_*), suggesting that plants were facing phytotoxicity but were able to cope with it (Araniti et al., 2018b).

Similarly, recent studies assayed rutin, a glycoside combining the flavonol quercetin and the disaccharide rutinose, on the model species *A. thaliana*. This metabolite, particularly present in the invasive species *Acacia melanoxylon*, affected the photosynthesis and excitation energy flux responses (Hussain and Reigosa, 2016). Hussain and Reigosa observed that the dark-adapted PSII (*F_*v*_/F_*m*_*) and the non-photochemical quenching Φ*_*NPQ*_* were significantly inhibited after 7 d of treatment. Moreover, plant response to rutin was characterized by a marked decrease in excitation energy fluxes (Φ*_*II*_*, non-photochemical quenching coefficient (qN) and Φ*_*NPQ*_*], suggesting plant damage.

The aromatic organic compound coumarin has been found to indirectly affect the photosynthetic machinery through induction of ROS burst and inhibition of several enzymes involved in ROS scavenging activity (Araniti et al., 2017d). Authors demonstrated, through an integrated physiological and *-omic* approach, that coumarin severely inhibited the effective quantum yield of the PSII, the maximum PSII efficiency, the energy dissipation in the form of heat, the estimated electron transport rate and the coefficient of the photochemical quenching. On the other hand, it significantly stimulated both the fluorescence emission and the coefficient of the non-photochemical quenching.

The identification of the mode of action of a molecule, both synthetic and natural, is a delicate process that needs to exclude from the experimental conditions all the sources of variability (e.g., lack of nutrients, temperature changes, light variation, etc.). CF could be affected by a wide number of both biotic and abiotic factors (e.g., drought, heat, flooding, temperature variation, parasitization, etc.) (Dong et al., 2020; Duarte et al., 2020; Zhou et al., 2020; Zhuang et al., 2020), which makes the evaluation of the mode of action of natural molecules quite impossible because of the overlapping of stressing conditions, which makes impossible to attribute the observed effects to the molecule assayed. Therefore, it is strongly suggested to perform experiments in completely controlled conditions using phytotrons and cropping plants hydroponically or on inert substrates enriched with nutrients and carbon sources (Graña et al., 2013b, 2016; Araniti et al., 2017c). Once identified the target and the potential mode of action of the molecule, the experiments could move to the next step, which consists in bioassays in microcosms trying to mimic natural conditions in greenhouses and/or open fields. This will allow the identification of the effectiveness of the molecules and their impact on the environment (e.g., on soil microorganisms) (Jouini et al., 2020). Barbagallo et al. (2003) used the CFI technique to rapidly screen the leaves metabolic perturbations induced by six different herbicides. However, although the same parameters are used to detect synthetic herbicides effects, those effects are not necessarily similar to natural bioherbicides, especially when secondary metabolites are known to commonly show more than one mode of action.

## Physiological Interpretation of Measured Parameters by Imaging Fluorescence

Multiple combinations of CFI parameters can be found in a leaf when exposed to a stressing factor (Table 2). Therefore, a correct physiological interpretation of the decreases and increases of the different parameters will give information about the ability of the plant to face stress with efficient protection mechanisms or with more toxic and inefficient dissipation mechanisms, which will lead to plant damage and stress. Increases of heat or fluorescence emission can occur in the plant without significantly reduce the PSII photochemical efficiency, (Φ*_*II*_*), as some types of stress do not instantly affect PSII quantum yield (Lopes et al., 2012). Heat increases are sometimes compensated by decreases in fluorescence emission and the other way around (scenarios 1–2, Table 2), because the stress is not strong enough, and effective compensation occurs in the electron transport chain, or because the measurements were recorded in an early phase of the stress response, before damage can be detected.

**TABLE 2:** Relevant parameters to be considered in the physiological interpretation of the measurement of chlorophyll *a* fluorescence.

	**Physiological interpretation**	**Φ*_*II*_***	**Φ*_*NPQ*_***	**Φ*_*NO*_***	***F_*v*_/F_*m*_***	**ETR**
1	Plant copes stressing situation by enhancing regulated energy dissipation. No damages are detected.	=	↑	= /↓	=	=
2	Plant copes stressing situation by enhancing limited fluorescence emission. No damages are detected.	=	= /↓	↑	=	=
3	Plant copes stressing situation by enhancing regulated energy dissipation, but PSII photosystem efficiency is significantly reduced, although ETR remains unchanged. Dynamic photoinhibition starts in the plant.	↓	↑	=	=	=
4	Plant faces stressing situation by enhancing regulated energy dissipation, but this strategy is not enough and fluorescence emission increases. Photosynthetic efficiency is significantly reduced and dynamic photoinhibition occurs.	↓	↑	↑	=	=
5	Plant copes stressing situation by enhancing regulated energy dissipation, but PSII photosystem efficiency and electron transport rate are significantly reduced. Plant is under dynamic photoinhibition.	↓	↑	↑	=	↓
6	Stressing situation reduces photosynthetic efficiency, but the plant cannot enhance regulated energy dissipation and heat emission remains unchanged or even decreases, increasing fluorescence emission. Plant is not damaged yet, but could be in brief.	↓	= /↓	↑	=	=
7	Stressing situation reduces photosynthetic efficiency, increases fluorescence emission and the plant cannot enhance regulated energy dissipation (heat). This situation reduces electron transport rate, but no physical damage is detected in the antennae complex, i.e., photoprotective mechanisms are efficient.	= /↓	= /↓	↑	=	↓
8	Stressing situation reduces photosynthetic efficiency, the plant cannot enhance regulated energy dissipation and fluorescence emission increases. This situation induces physical damage in the antennae complex and chronic photoinhibition occurs. Photoprotective mechanisms are not efficient enough due to an excess of excitation energy.	↓	= /↑/↓	↑	↓	=
9	Stressing situation reduces photosynthetic efficiency, increases fluorescence emission and the plant cannot enhance regulated energy dissipation (heat). This situation induces physical damage in the antennae complex and reduces electron transport rate. Plant undergoes chronic photoinhibition.	↓	= /↓	↑	↓	↓
10	Electron transport rate and photosynthetic efficiency are significantly decreased under stress. This could be induced by an alteration on the biochemical phase of the photosynthesis and results in altered photochemical quenching. Excess energy can be emitted under regulated (heat) or non-regulated (fluorescence) mechanisms.	↓	= /↓	= /↑	=	↓
11	Photosynthetic efficiency increases under stress to respond to the energetic demand required to synthesize molecules involved in the response to stress.	↑	= /↓	=	=	= /↑

		**Φ*_*II*_***	**Φ*_*NPQ*_***	**Φ*_*NO*_***	**qN**	**qP**

12	An increase of qN without increase of Φ*_*NPQ*_* suggests that plants are experiencing photoinhibition or about to undergo it	= /↓	=	= /↑	↑	= /↓
13	A reduction in qP suggests physical damage at the PSII reaction centers	↓	= /↑	= /↑	= /↑	↓

Decreases of Φ*_*II*_* usually result in increases of non-photochemical quenching (heat) when the plant is able to orchestrate regulated energy dissipation through xanthophylls cycle, pH regulation, or reversible phosphorylation of light harvesting chlorophyll *a/b* binding proteins (LHCII) (scenarios 3–5, Table 2). By contrary, Φ*_*II*_* decreases will result in increases of non-regulated energy emission when photochemical energy conversion fails, and protective regulatory mechanisms are inefficient (scenarios 4–9). However, increases of Φ*_*NO*_* and decreases of Φ*_*II*_* does not necessarily indicate that the plant is already damaged, as might be being protected, albeit weakly, until further radiation occurs or stressing factor remains (scenarios 4–5). The latter was shown by Martínez-Peñalver et al. (2012) with the secondary metabolite protocatechualdehyde, where strong increases in Φ*_*NO*_* didn’t result in ETR or *F_*v*_/F_*m*_* decreases, or by Araniti et al. (2018b) in *Zea mays* seedlings treated with *trans*-cinnamic. Ferredoxin or Mehler reaction can be accepting the electrons diverted from photochemical reactions when no decreases in ETR are observed (scenario 4). The same values of Φ*_*NPQ*_*, Φ*_*NO*_*, Φ*_*II*_*, and *F_*v*_/F_*m*_* are observed in scenario 5, although decreases in ETR also occurred related to decreases in the efficiency of PSII.

Scenarios 4–7 of Table 2 show dynamic photoinhibition, where decreases of photochemical conversion occur and plant has serious problems to cope with the incident radiation, but no physical damage is detected at the photosynthetic apparatus. The latter is suggested by unchanged or very slight decreased *F_*v*_/F_*m*_* values, which reveal flexible adjustment to environmental conditions (Werner et al., 2002). In contrast, plants showing statistically significant decreases in PSII efficiency (Φ*_*II*_*) and strong increases in fluorescence emission (Φ*_*NO*_*) usually show weak to strong decreases in the maximum PSII efficiency (*F_*v*_/F_*m*_*), which suggests different degrees of physical damage at the protein-pigment complexes of the light harvesting antennae of PSII (scenarios 8–9, Table 2). Consequently, chronic photoinhibition due to acute or long-term environmental stress occurs, as previously found for some natural compounds as citral or *trans*-caryophyllene (Graña et al., 2013b; Araniti et al., 2017c). Late *F_*v*_/F_*m*_* decreases can suggest a secondary effect of the stressing factor; i.e., Sánchez-Moreiras et al. (2011) demonstrated that *F_*v*_/F_*m*_* and Φ*_*II*_* reduced values appeared to be correlated to necrotic processes after secondary oxidative stress, consequence of early senescence processes.

On the other hand (Table 2, scenario 9), plants could be unable to develop strategies of protection (as heat emission) when a stress factor has a direct effect on the photosynthetic machinery, increasing non-regulated emission of energy as fluorescence and inducing photoinhibition. Then, biochemical and photochemical phases of photosynthesis can be affected, altering electron transport and damaging PSII reaction centers, as found by Sánchez-Moreiras et al. (2011) in leaves of *Arabidopsis* plants treated with the secondary metabolite BOA.

Sometimes, similar values of Φ*_*II*_*, Φ*_*NPQ*_*, and Φ*_*NO*_*, and ETR result in totally different impact on the maximum PSII efficiency (*F_*v*_/F_*m*_*), as previously found by Díaz-Tielas et al. (2014). Authors demonstrated in this study that, although similar values of PSII quantum yield, heat dissipation, fluorescence emission and electron transport rate were found for *Arabidopsis* plants sprayed or watered with the flavonoid *trans*-chalcone, watered plants were able to protect the antennae complex and the reactions centers along the whole experiment, while sprayed plants showed decreased values of *F_*v*_/F_*m*_* at the end of the experiment, suggesting underprotection and possible physical damage at the antennae complex.

All of these scenarios (1 – 9) are usually found when the natural product affects photochemical phase first, by altering antennae complex, reaction centers, pH gradient through the thylakoid membranes, or competing with other molecules to accept the electrons coming from PSII and PSI. However, in some cases, i.e., senescence processes, stomatal closure or other situations restricting CO_2_ assimilation, is the biochemical phase the first part affected of the photosynthetic machinery (Ping et al., 2015), resulting in decreases of electron transport rate (ETR values) without or with slight alterations of the other parameters (scenario 10). When accumulated ROS cannot be detoxified (Paul and Foyer, 2001), and antioxidant systems or other photo-protective mechanisms, as xanthophyll cycle or increased photorespiration, are not enough, physical damages at the PSII reaction centers or antennae complexes can occur, resulting in a scenario similar to scenario 9 of Table 2. This has been recently found for plants treated with the nitrogen-containing natural compound norharmane (López-González et al., 2020).

On the other hand, the necessity of synthesizing new molecules in response to adverse conditions (i.e., proline, antioxidants, and others; Gonzáles et al., 2008) can lead to increased photosynthetic efficiency in plants (Desotgiu et al., 2012; scenario 11, Table 2). This increase in operating efficiency (Φ*_*II*_*) can be usually obtained by reducing regulated dissipation of light energy (Φ*_*NPQ*_*) as shown by Martínez-Peñalver et al. (2012) in plants treated with 3,4-dihydroxybenzaldehyde, which found a compensatory increase of Φ*_*II*_* at the beginning of the experiment that was correlated with a decrease of Φ*_*NPQ*_*.

Coefficients qN and qP (scenarios 12 and 13, Table 2), can give more information about non-photochemical quenching and status of the PSII reaction centers, making data interpretation easier. The increase of qN without Φ*_*NPQ*_* increase (scenario 12) suggests that plants are experiencing photoinhibition or about to undergo it. The same for the combination shown in scenario 13, where stressing situation reduced the coefficient qP, suggesting a physical damage at the light-harvesting complex II (LHCII), which could firstly occur at the site of PSII reaction center that is associated with PSII electron transport (inhibiting PSII repair), at the water splitting site in the oxygen evolving complex (OEC), or at both sites at the same time (Oguchi et al., 2009, 2011; Zavafer et al., 2015). Similar results were found by Araniti et al. (2017c) in plants treated with an intermediate concentration of *trans*-caryophyllene or by Gao et al. (2018), when testing different secondary metabolites, such as usnic, BA, CA, and SAs.

Finally, Φ*_*NPQ*_*/Φ*_*NO*_* ratio; i.e., the ratio of quantum yield of regulated to non-regulated energy dissipation in PSII, which is connected to the ability of photosynthetic apparatus to activate photoprotective mechanisms (Klughammer and Schreiber, 2008), can tell in a rapid and easy way if the plant is photo-protected (high values) or is undergoing photoinhibition or photodamage processes (low values).

However, all these data cannot provide direct information about where the damage is occurring first, or how the acclimation responses are being implemented into the leaf. Moreover, the so-called phenomenon of “patchy photosynthesis” due to ununiform stomatal closure or to heterogeneities in the leaves (Meyer and Genty, 1999; Beyschlag and Eckstein, 2001; Lopes et al., 2012) can be a source of errors when recording improperly single-point CF measurements. Imaging techniques avoid misunderstandings due to this phenomenon, as leaf fluorescence is obtained at once (Martínez-Peñalver et al., 2012).

**FIGURE 1:**
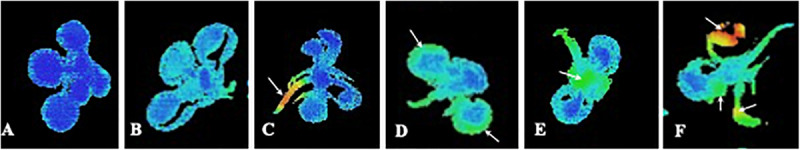
Imaging pictures of maximum PSII efficiency (*F_*v*_/F_*m*_*) of *Arabidopsis* seedlings at different physiological status. **(A)** Control plant with optimal PSII photosynthetic efficiency; **(B)** Whole decreased of photosynthetic efficiency that is homogenous all over the plant; **(C)** Decreased photosynthetic efficiency in the cells close to the vascular bundles. The rest of the plant remains intact; **(D)** Strong decreased of photosynthetic efficiency in the border of the older leaves; **(E)** Decreased photosynthetic efficiency mainly in the younger leaves; and **(F)** Strong decrease of photosynthetic efficiency in the whole plant, with punctual inhibition spots in the leaves.

Unfortunately, very few studies have analyzed the whole plant fluorescence emission after natural compound phytotoxicity, even though CFI allows this measurement in an easy and fast way. Therefore, with this paper, we want to encourage researchers in phytotoxicity to record the whole response of the plant and monitoring this response all over the time to obtain a more accurate information about the effect of the compound. Careful spatial analysis of the images of fluorescence emission is appropriate when studying phytotoxic effects in plants, as far as we do not know, before measuring fluorescence, which type of effect will be induced in the plant. This is applicable to sprayed but also to watered application of the compounds, since effects related to some types of stress induced by the compound (i.e., oxidative or water stress) can result in spots of fluorescence emission in the leaves of the treated plants. Figure 1A shows an *Arabidopsis* seedling grown under optimal conditions of light, temperature, humidity and CO_2_ concentration. As seen, *F_*v*_/F_*m*_* values are optimal and homogeneous all over the plant. However, some stressing factors, as plant natural compound-induced phytotoxicity, can decrease the overall photosynthetic capacity of the plant decreasing maximum PSII efficiency in young and old leaves, as shown in Figure 1B. In this case, the homogeneous distribution of the effect makes single-point measurements of fluorescence precise and accurate, anyway if measurements are done in young or old leaves, or in the center or the border of the leaves. By contrary, some natural compounds induce asymmetric effects on the photosynthetic capacity of the plant (Sánchez-Moreiras et al., 2011; Graña et al., 2013b), as shown in the rest of the images included in Figures 1C–F.

An asymmetric effect starting in the cells closed to the vascular bundles is shown in Figure 1C, as indicated by orange and red colors related to decreased *F_*v*_/F_*m*_* values. This effect could be related to early water stress phenomenon, as previously shown in thermal and salt stress by Martínez-Peñalver et al. (2011), which found a characteristic pattern of reduction that affected the central areas of the young leaves close to the vascular bundles. This localized increase of fluorescence could be due to altered water stress status after treatment with a natural product, or to an abscisic acid mimic action of the natural product applied, as suggested by Graña et al. (2013b) when studying the effects of the monoterpenoid citral.

A different asymmetric effect is shown in Figure 1D. In this case, greater fluorescence emission is localized at the edges of the older leaves, while the vascular bundles and the youngest leaves in the rosette remain intact. Different studies have indicated that this pattern could be reminiscent of early senescence as suggested by the images of fluorescence distribution in *Arabidopsis* plants treated with citral (Graña et al., 2013b) or with the secondary metabolite *o*-coumaric acid (Zheng et al., 2012).

Figure 1E shows an unusual distribution of reduced maximum PSII efficiency, with lower values in younger than in older leaves. This scenario could be the case of a natural product inducing a strong effect on target sites directly related to the photosynthetic apparatus. This effect could result in a stronger damage on the fully synthesizing leaves, as that seen by Díaz-Tielas et al. (2017) in chalcone-treated seedlings, which induced early plasma membrane depolarization and dramatic effects on chloroplasts structure and function.

Finally, Figure 1F shows an image where heterogeneous damage appears indistinctly for young or old leaves. Arrows point out damaged spots in the different leaves. As indicated by orange and red color, some parts of the leaves remain intact while others are extremely damaged. This effect has been previously related to oxidative stress and necrotic process in Arabidopsis plants treated with different natural compounds (Martínez-Peñalver et al., 2011; Sánchez-Moreiras et al., 2011). In this way, Araniti et al. (2017b) demonstrated overlapping of ROS accumulation in the leaf and the area characterized by *F*_*v*_/*F*_*m*_ reduction when lettuce was treated with volatiles released by *Dittrichia viscosa*. Moreover, this isolated inhibition spots (increased fluorescence emission) could be also found when the studied natural compound is sprayed to the leaves and behaves as a PSII inhibitor directly binding to the D protein and interrupting the electron transfer chain of PSII re-emitting the excitation energy as fluorescence, which will result in an easily discernable decrease of F_*v*_/F_*m*_ values, as has been previously found for synthetic herbicides (Muller et al., 2008; Wang et al., 2018). This decrease will happen first in the areas of contact of the sprayed compound with the leaf, resulting in isolated spots of increased fluorescence emission.

## Conclusion

As reviewed at the beginning of this paper, and analyzed in the physiological interpretations of the images obtained, imaging fluorescence analyses of the whole affected plant provides accurate and fundamental information for the interpretation of plant response to stress not provided by single-point techniques or analyses of photosynthetic parameters independently analyzed of their spatial distribution in the plant. Monitoring imaging fluorescence can help to understand how damage evolves and how response is orchestrated into the plants.

Because of the lack of information and the restricted use of this technique in the field of natural-herbicide discovery, research efforts should be focused on creating a common procedure to study the mode of action of natural compounds, without avoiding protocol innovations. The use of a model sensitive species, such as *A. thaliana*, could allow the standardization of the method, which could be used worldwide allowing the reproducibility of the data. This could allow the creation of a database of the signatures of the CF and the trend of the various parameters in response to the treatments. In addition, this technique could be further used as a sensitive tool to identify the effective doses (stimulatory and inhibitory) of the natural compounds as alternative or support to the classical dose-responses curve.

## Author Contributions

AS-M: idea and writing and editing. FA: writing and reviewing. EG: image acquisition and editing. MR: idea and reviewing. All authors contributed to the article and approved the submitted version.

## Conflict of Interest

The authors declare that the research was conducted in the absence of any commercial or financial relationships that could be construed as a potential conflict of interest.
